# Bayesian analysis in confirmatory clinical trials: A narrative review and discussion of current practice

**DOI:** 10.1177/17407745261437669

**Published:** 2026-04-07

**Authors:** Rebecca M Turner, Conor D Tweed, Trinh Duong, Deborah Ford, Michelle N Clements, Mahesh KB Parmar, Anna Turkova, Ian R White

**Affiliations:** 1 MRC Clinical Trials Unit, University College London, London, UK

**Keywords:** Bayesian analysis, confirmatory trials, phase III trials, review

## Abstract

**Background:**

Bayesian methods allow trial investigators to combine evidence obtained within a clinical trial with relevant evidence that is available outside the trial. Bayesian analyses are now widely used in the drug development process, to inform internal ‘go/no-go’ decisions about planned studies, for example when deciding whether a drug should proceed from phase II to a phase III trial. However, Bayesian analyses are not commonly used for analysis of phase III (confirmatory) trials.

**Methods:**

In this article, we performed a narrative review of confirmatory trials using Bayesian methods for their primary analysis, to explore which types of trials chose Bayesian methods, why they chose a Bayesian analysis and how the methods were used. We reviewed published papers over a 6-year period and explored the characteristics of trials using Bayesian methods for their primary analysis, their reasons for choosing a Bayesian analysis, whether any informative priors were used and if so how they were informed. Next, we selected four trials from the review as case studies and presented their motivation for using Bayesian methods and their Bayesian analyses in more detail.

**Results:**

Our narrative review found that the number of Bayesian methods in confirmatory clinical trials has approximately doubled over the past 6 years, reflecting growing familiarity among investigators. Ninety-four papers were eligible for inclusion, presenting results from 69 separate trials. The most common reason given for choosing Bayesian methods was to make direct probability statements about the superiority and/or futility of the interventions evaluated; this was mentioned for 49% of trials. Flexibility in adapting the design or use of Bayesian stopping rules was another very common motivation, cited for 47% of trials. Borrowing information through informative priors was cited for a much smaller proportion (16%) of trials. The majority of trials (75%) specified vague or weakly informative priors for all parameters.

**Conclusion:**

Among the reasons given for choosing Bayesian methods, we consider the use of informative priors or making direct probability statements to be the strongest motivations for a Bayesian analysis, because there are no equivalent frequentist approaches. Making direct probability statements was the most common motivation provided, while informative priors were not often used. In settings with recruitment difficulties, we recommend considering borrowing relevant information, to gain power and precision. In all confirmatory trial settings, we recommend that Bayesian approaches are used only with careful justification, investigators make clear whether the methods and priors were pre-planned, and alternative frequentist approaches are considered.

## Background

Bayesian methods for analysis of clinical trials have been widely discussed in the statistical and medical literature. Bayesian statistical inference provides a formal framework for combining evidence obtained within the trial with relevant evidence that is available outside the trial and offers potential advantages over frequentist analysis.^1,2^ Borrowing evidence from external sources can be beneficial in settings such as rare diseases or paediatric populations, where performing large-scale trials can be very difficult, or sometimes impossible.^3^ Trial results obtained from a Bayesian analysis can be expressed using probabilistic language, for example by reporting the probability that the experimental treatment was superior to the control treatment, which may be viewed as more intuitive and easier to interpret than confidence intervals and *p* values. Predictions are easily made from a Bayesian analysis, meaning that investigators can predict the probability of achieving a significant result in a planned trial given previous data or in an ongoing trial given interim data, and base decisions on these probabilities.^4^

Alongside the advantages of taking a Bayesian rather than frequentist approach to analysis of a trial, there are potential disadvantages to consider. Informative prior distributions based on opinion or data must be carefully chosen and justified, and may be criticised later by reviewers or readers for not reflecting the views of all relevant groups. Vague prior distributions representing lack of prior knowledge should also be chosen carefully because results can be sensitive to choice of vague prior, particularly for parameters informed by sparse data.^5^ Ideally, a Bayesian analysis should be repeated using two or more different priors, to assess the sensitivity of posterior inferences.^1^ If an informative prior is used, sensitivity can be explored by varying the weight allocated to the external data or opinion. Most trial statisticians have substantially more experience and training in the use of frequentist methods, meaning that errors in implementation may be more likely.^6^ Trial investigators may be concerned that Bayesian results are less likely to be accepted by regulators or policy makers, because of a lack of familiarity and the limited regulatory guidance available on Bayesian approaches.^6–8^ There is also a danger that Bayesian methods appear opaque and cannot be followed or easily reproduced by others not involved in the analysis.

Bayesian analyses are now widely used in the drug development process, to inform internal ‘go/no-go’ decisions about planned studies, for example when deciding whether a drug should proceed from phase II to phase III trial.^9^ However, Bayesian analyses are still not commonly used for analysis of phase III (confirmatory) trials.^6^ The aims of this article are to review the recent use of Bayesian analyses in confirmatory trials, to explore which types of trial have chosen Bayesian methods and how they were used. We have reviewed published papers over a 6-year period from 2019 to 2024 and explored the characteristics of trials using Bayesian methods for their primary analysis, why a Bayesian approach was chosen, whether any informative priors were used and if so how they were informed. Next, we selected four trials from the review as case studies and presented their motivation for using Bayesian methods and their Bayesian analyses in more detail.

## Methods

We carried out a narrative review to identify confirmatory (or phase III) trials in which Bayesian methods had been used for the primary analysis of the primary outcome. The aims of the review were to find out how often informative priors were used and for which parameters, what type of justification is provided for the choice of informative priors, and the reasons authors give for using Bayesian methods (if any). We also wanted to explore the characteristics of confirmatory trials choosing Bayesian methods for their primary analysis, for example, trial design types, clinical focus and types of interventions evaluated.

Our inclusion criteria were as follows: (1) randomised trial evaluating a healthcare intervention, (2) Bayesian methods used for the primary analysis of the primary outcome and (3) results paper published between 2019 and 2024. Our focus was on confirmatory trials that were designed with the aim of producing results that would influence practice. We, therefore, excluded trials that were described as exploratory, phase II, phase I or a pilot study and trials that didn’t provide justification of their chosen sample size with respect to power, precision or posterior probability. We carried out a search on PubMed using the following search terms:

(bayes*[Title/Abstract] OR (posterior probabilit*[Title/Abstract]) OR (credible interval[Title/Abstract])) AND (clinical trial[pt]) NOT ((phase 2[Title/Abstract]) OR (phase II[Title/Abstract]) OR (phase 1[Title/Abstract]) OR (phase I[Title/Abstract]) OR meta-analysis[Title/Abstract]) AND 2019/1/1:2024/12/31[dp]

The abstracts of all papers matching the search criteria were screened for relevance. Full texts of manuscripts were subsequently reviewed for papers that were judged potentially relevant on the basis of the abstract. From papers that were eligible for inclusion, we extracted trial design type, medical area, intervention type, primary outcome, funding source, reasons given for using Bayesian methods, whether any informative priors were specified and which parameters they were specified for, justification for choice of informative priors and analysis model.

From the included trials, we selected four trials which gave different reasons for using Bayesian methods and explored these in more detail as case studies.

## Results

### Results from narrative review

Our search identified 808 papers; we screened their abstracts and identified 192 papers as potentially relevant. Based on subsequent review of full texts, 94 papers were judged to be eligible for inclusion. Some trials had published multiple results papers during our inclusion period; in total, papers from 69 separate trials were included (Supplemental material).

**Figure 1.**
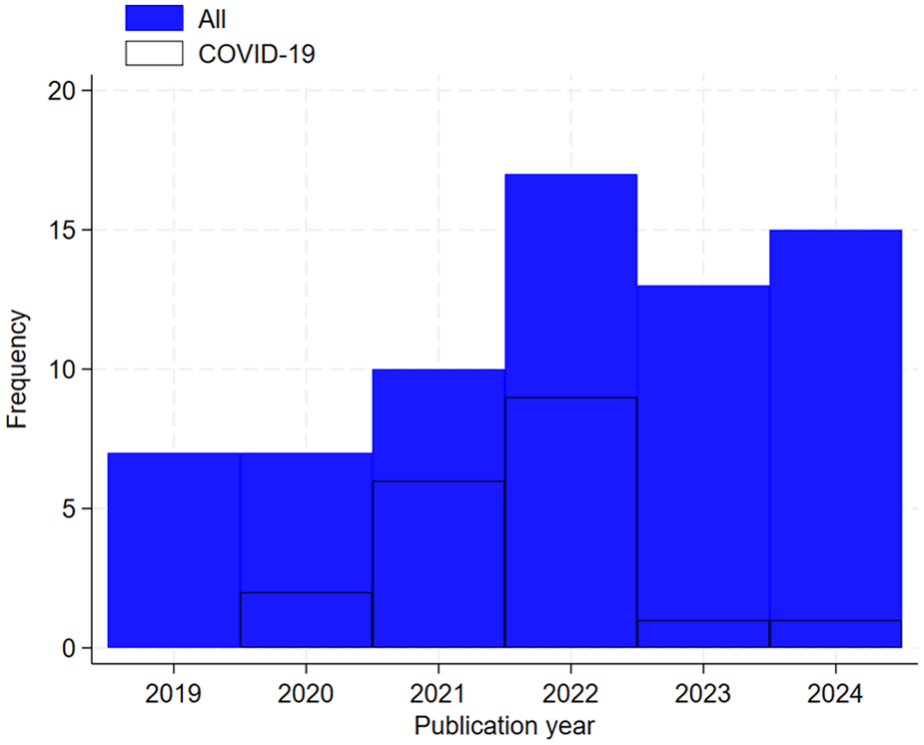
Publication year of included trials, overall and for trials in COVID-19.

The characteristics of the included trials are presented in Table 1. The frequency of eligible trials doubled over time, from 7 in 2019 to 15 in 2024; in 2021–2022, a large proportion of the eligible trials were in COVID-19 (Figure 1). The most common single reason for choosing Bayesian methods for analysis was that researchers wanted to make direct probability statements about superiority and/or futility of the interventions evaluated (Table 2). This reason was mentioned for 49% of trials. The next most common reasons were flexibility in adapting the trial design and use of Bayesian stopping rules. We note that three adaptive platform trials did not explicitly state their reason(s) for using Bayesian methods, but they used Bayesian decision rules throughout and the statisticians involved have elsewhere discussed preferring a Bayesian approach primarily for the flexibility it provides for complex adaptive designs.^10^ We have grouped these similar reasons together as ‘using a flexible adaptive design’, which was mentioned for 47% of trials in total. For a much smaller proportion of trials (16%), borrowing information through informative priors was mentioned as one of their reasons for choosing a Bayesian analysis. Computational reasons were cited for 12% of trials, for example, where the estimation method for the pre-defined frequentist analysis became unstable and Bayesian analysis was used as an unplanned alternative method, or where a Bayesian approach offered more flexibility in modelling. For two trials (4%), handling missing data was mentioned as one of their motivating reasons: one used Bayesian methods for imputing missing data, while the other chose Bayesian estimation to reduce potential biases from losses to follow-up. Of 69 included trials, 12 (17%) did not give any reasons for choosing a Bayesian analysis. In Table 3, we provide more details about the justifications given in the included trials, for each of the four most common reasons for choosing Bayesian analyses, and we suggest some alternatives to a Bayesian approach that could be considered.

**Table 1.** Characteristics of included trials.

Medical area	Frequency (%) (*n* = 69)
Infectious diseases	26 (38%)
Mental health/behavioural	12 (17%)
Cardiovascular	11 (16%)
Central nervous system	6 (9%)
Cancer	3 (4%)
Respiratory	3 (4%)
Musculoskeletal	3 (4%)
Gynaecology/pregnancy/birth	2 (3%)
Autoimmune diseases	2 (3%)
Other (inguinal hernia in preterm infants)	1 (2%)
Design	Frequency (%)
Parallel arms with individual randomisation	46 (67%)
Platform^a^	12 (17%)
Parallel arms with cluster randomisation	6 (9%)
Stepped wedge	2 (3%)
Cross-over	2 (3%)
Other (multiplatform: integration of three platform trials)	1 (2%)
Intervention type (experimental/first)	Frequency (%)
Pharmacological	33 (48%)
Psychosocial/educational/behavioural	20 (29%)
Surgical	8 (12%)
Medical device	5 (7%)
Complex	2 (3%)
Other (outdoor spraying for malaria control)	1 (2%)
Funding source	Frequency (%)
Not-for-profit sponsor(s)	46 (67%)
For-profit sponsor(s)	17 (25%)
Both for-profit and not-for-profit sponsor(s)	6 (9%)

a Defined as a randomised adaptive trial, with the potential to compare multiple interventions, which can evolve over time by addition or removal of treatment arms.

**Table 2.** Reasons for choosing Bayesian methods for primary analysis, where stated.

Reasons given	Frequency (%)^a^ (*n* = 57)
Making direct probability statements	28 (49%)
Using a flexible adaptive design (e.g. Bayesian stopping rules and flexible interim analyses)	27 (47%)
Borrowing information	9 (16%)
Computational reasons	7 (12%)
Handling missing data	2 (4%)

a Of 69 included trials, 12 (17%) did not provide their reason(s) for choosing a Bayesian analysis. Some trials gave more than one reason.

**Table 3.** Issues to consider when choosing whether to use a Bayesian approach.

Reason for choosing Bayesian approach	Advantages of Bayesian approach (cited by trials included in our review)	Alternatives to Bayesian approach
Making direct probability statements	• Accessible • Clinically meaningful • Useful for decision-making • Outputs can inform an economic analysis	Convert a frequentist result into posterior probabilities, as in the EURO EWING trial.^11^
Using a flexible adaptive design	• Flexibility in the timing of interim analyses • Rapid decision-making • Keeping the design flexible • Allowing the observed data to determine the appropriate sample size	Frequentist adaptive platform designs also allow early stopping of arms and addition of new arms. An advantage is that power and type I error are more easily controlled, and trial results may be more likely to be accepted by regulators.
Borrowing information from an external source or internally within the trial	• Reducing the required sample size of planned trial • Increasing power and precision • Addressing recruitment difficulties in an ongoing trial	Where recruitment is difficult, other options for making a trial feasible could be to relax the significance level above the conventional 5%,^8^ or change to a more information-rich outcome.^12^ Alternatively, a meta-analysis approach could be used to combine previous data with data from a new trial.
Computational reasons	• Alternative approach when planned frequentist analysis had failed (e.g. convergence problems or singularity errors) • Greater flexibility in modelling	A simpler frequentist analysis could be considered, if the implementation difficulties are caused by model complexity.

To examine whether the motivation for choosing Bayesian methods varied across medical areas, we looked at the three largest medical areas separately and grouped the smaller areas together. There was some evidence that citing the motivation to use a flexible adaptive design varied across areas: this was mentioned by 6/11 (55%) cardiovascular trials, 17/26 (65%) infectious disease trials, 1/12 (8%) mental health/behavioural trials and 3/20 (15%) other trials. The majority of infectious disease trials were in COVID-19, and 74% of these trials cited the motivation of using a flexible adaptive design. If the COVID-19 trials are excluded, the variation across medical areas is less pronounced, since 43% of the remaining infectious disease trials mentioned using a flexible adaptive design.

**Figure 2.**
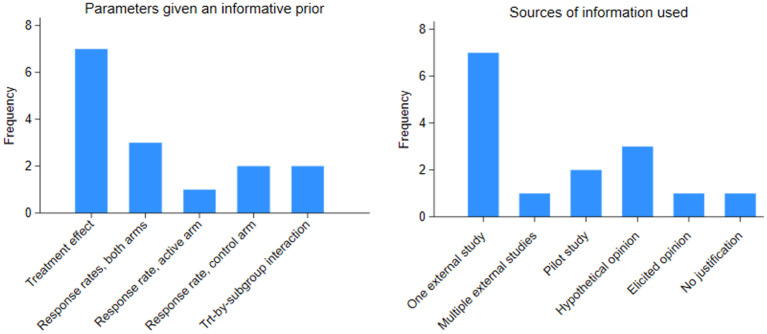
Frequencies of types of parameter given informative priors and the sources of information used to construct informative priors.

Among 57 trials where the description of their priors was sufficiently clear, the majority (75%) had specified vague or weakly informative priors for all parameters. Choices made for vague priors were rarely justified. For 10/69 trials, the priors were not described clearly enough to determine whether any parameters were given an informative prior. Among the 15 trials that specified at least one informative prior, 7 trials specified an informative prior for the treatment effect. The remainder specified informative priors for the response rate in each arm (three trials), response rate in the active arm (one trial), response rate in the control arm (two trials) and a treatment-by-subgroup interaction (two trials). Figure 2 shows the frequencies of the parameters given informative priors and the sources of information on which these were based. Of the 10 trials borrowing from external data, 4 trials down-weighted the borrowed data dynamically according to the degree of discrepancy with the observed data, 2 trials down-weighted the borrowed data by a fixed amount and 4 trials did not down-weight the borrowed data. Dynamic borrowing was implemented using robust mixture priors (one trial), power priors (two trials) or hierarchical modelling (one trial). Borrowing was pre-planned in most cases (seven trials), while in one trial, a borrowing analysis was chosen partway through to address recruitment difficulties,^13^ and for two trials, it was unclear whether borrowing was pre-planned. Three trials constructed an informative prior to represent a hypothetical point of view: for two trials, this was a sceptical prior indicating that there was likely to be no difference between arms; for the other, it represented the view that all subgroups were likely to have a similar treatment effect. One trial constructed a prior for a treatment-by-subgroup interaction based on eliciting opinions about the difference between subgroups. One trial specified an informative prior for the control arm based on the expected response rate, but did not give further justification. Of the 15 trials that specified at least one informative prior, 4 trials also presented a sensitivity analysis using alternative priors; in 3 cases, the sensitivity analysis used vague priors for all parameters, while 1 trial presented results based on an alternative informative prior.

### Case studies of confirmatory trials using a Bayesian approach

#### BIOSTEMI

The BIOSTEMI trial^14^ (Table 4) used Bayesian methods to borrow information from an earlier trial (BIOSCIENCE),^15^ which had compared the same interventions; the population of BIOSTEMI matched a pre-specified subgroup in BIOSCIENCE in which superiority had been concluded for the experimental intervention. Robust mixture priors were planned to control the type I error rate by down-weighting the borrowed information according to discrepancy between the borrowed results and BIOSTEMI results, meaning the Bayesian results would differ from a standard meta-analysis pooling information from both studies.

**Table 4.** Design characteristics of case studies.

	BIOSTEMI	EURO EWING 2012	PRINCIPLE	ODYSSEY
Design	Parallel arms with individual randomisation	Parallel arms with individual randomisation	Platform trial	Parallel arms with individual randomisation
Population	Patients with acute ST-segment elevation myocardial infarction (STEMI) undergoing primary percutaneous coronary intervention	Patients aged 2–49 years with newly diagnosed Ewing sarcoma	People with symptoms of COVID-19, aged at least 65 years or 50–64 years with comorbidities	Children living with HIV aged 4 weeks or older and weighing at least 3 kg
Interventions compared	Biodegradable-polymer sirolimus-eluting stents vs. durable-polymer everolimus-eluting stents	Standard European chemotherapy regimen vs. standard US chemotherapy regimen	Drugs with potential for widespread and generally safe use in the community compared against usual care	Dolutegravir-based antiretroviral therapy vs. standard of care
Primary outcome	Target lesion failure at 2 years	Event-free survival at 3 years	Coprimary outcomes: time to first self-reported recovery within 28 days, and hospital admission or death within 28 days	Virological or clinical failure by 96 weeks
Planned sample size	1250 patients	600 patients	No fixed sample size	700 children in main cohort (weighing ≥14 kg); 60 children in 3–14 kg cohort
Method for sample size determination	Sample size was chosen to achieve 80% probability of declaring superiority in the planned Bayesian analysis, based on simulations.^16^	Posterior probabilities for one treatment being better or not more than 5% worse were explored for an achievable sample size (based on accrual rate) and a range of observed hazard ratios.	Trial planned to continue until either superiority or futility was declared for each treatment, or the COVID-19 pandemic expired. Superiority thresholds chosen in advance through simulation, to control type I error.	Sample size of main cohort provided 90% power to demonstrate non-inferiority (with a 10% margin). Sample size for 3–14 kg cohort was based on pharmacokinetic analysis (at least 20 children required in each of three weight bands).

The primary analysis was performed using a Bayesian log Poisson model, with robust mixture priors declared for event rates in each arm. Within each arm, the mixture weights allocated to the informative prior and a Normal (0, 9) prior (chosen to be vague) were updated according to similarity between the BIOSTEMI and BIOSCIENCE results; the prior weights were set to 0.5 for each component. The informative component is the posterior distribution of the log-rate in each arm obtained from the BIOSCIENCE trial. The posterior median for the rate ratio for target lesion failure at 2 years, comparing biodegradable stents to durable stents, was 0.58 (95% posterior credible interval: 0.40–0.84). The posterior probability of biodegradable being superior to durable stents was 99.8%. To explore sensitivity of the results, the investigators also analysed the BIOSTEMI data in a standalone analysis using vague priors, and this produced fairly similar results with the same conclusion of superiority, but with a wider 95% posterior credible interval as expected (posterior median: 0.62; 95% posterior credible interval: 0.40–0.96; posterior probability of superiority: 0.985). The trial concluded that biodegradable stents were superior. The use of a Bayesian approach enabled the BIOSTEMI investigators to reduce the required sample size of the new trial by 44% through making use of existing data from a previous similar trial, from 1111 per arm to 625 per arm.

#### EURO EWING 2012

In the EURO EWING 2012 trial (Table 4),^11^ the investigators chose a Bayesian approach because they were comparing two standard regimens already in widespread use and, therefore, judged that a less stringent decision criterion than the conventional frequentist approach would be appropriate for determining whether one regimen was better than the other. An informal survey found that the trial’s leading clinicians would be happy to accept one regimen as standard if there were an 80% chance that it was better than the other.

Bayesian analyses were implemented using a two-stage approach: a frequentist Cox regression model was fit in the first stage, with adjustment for stratification variables; next, the estimated log hazard ratio was assumed normally distributed with variance determined by the total number of events, and a Bayesian analysis with vague priors was performed (no alternative priors were used in sensitivity analyses). Event-free survival at 3 years was 61% for the European regimen and 67% for the US regimen, with the corresponding hazard ratio estimated as 0.71 (95% posterior credible interval: 0.55–0.92) in favour of the US regimen. The posterior probability that the hazard ratio was less than 1 was greater than 99%, and the probability that it was less than 0.8 was greater than 81%. The authors discussed the benefits of probabilities being more intuitive and easier for clinicians and patients to understand than *p* values, which may be misinterpreted.

#### PRINCIPLE

The PRINCIPLE trial (Table 4) was an adaptive platform trial evaluating multiple community treatments for people with suspected COVID-19.^17^ The investigators used a Bayesian hierarchical modelling approach to gain precision in analyses by including patients randomised to usual care before the active treatment arm was opened as well as patients randomised concurrently, while allowing for potential temporal drift in the event rate by adjusting for time interval and smoothing across time intervals.^18^ Priors were chosen to be vague, and no alternative priors were used in sensitivity analyses. During our review period, the PRINCIPLE trial published six results papers,^19–24^ each comparing a different active treatment to usual care. Inhaled budesonide and favipiravir both met the pre-specified superiority criterion for time to first recovery (posterior probability of superiority greater than 99%), but did not meet the superiority criterion for hospitalisations/deaths (posterior probability greater than 97.5%).^23,24^ Posterior probabilities that hospitalisation/deaths were lower than under usual care were 96% for budesonide and 51% for favipiravir. Four other treatments were each stopped for futility: azithromycin, colchicine, doxycycline and ivermectin.^19–22^

The authors discussed their motivation for using a Bayesian modelling approach to allow inclusion of historical, nonconcurrent controls, while adjusting for changes in the control population over time, potentially increasing the precision of estimates and allowing stopping decisions to be made earlier. However, results from this time-adjusted analysis could potentially be biased when incorporating nonconcurrent controls in a platform trial, if underlying event rates differ between cohorts of participants with different sets of treatments available for randomisation.^25^ The clinical setting, standard of care and circulating variants changed rapidly during the COVID-19 pandemic and it, therefore, seems likely that underlying event rates varied between cohorts. In this setting, adjustment for cohort effects rather than time effects alone has been recommended, if nonconcurrent controls are included in the analysis. In the PRINCIPLE trial, sensitivity analyses were performed using only concurrent controls.

#### ODYSSEY

ODYSSEY was a non-inferiority trial evaluating dolutegravir-based antiretroviral therapy in children living with HIV (Table 4). The main trial recruited 707 children weighing ≥14 kg; 85 children weighing 3–14 kg were recruited 12 months later following a pharmacokinetics study. The investigators chose not to delay reporting results from the main trial population, meaning results from the younger children would be reported separately. However, a standalone analysis of the younger children would be inadequately powered due to the small sample size. Since treatment effects were expected to be similar across age groups, the investigators decided to use Bayesian methods to borrow information from the older children when analysing the younger children.

An interaction parameter was used to model the difference between treatment effects in the two cohorts. Elicitation of clinical opinions provided a prior distribution for the interaction, to inform the degree of borrowing; this was obtained before results from either cohort were available. In the primary Bayesian analysis, the estimated difference in virological or clinical failure in younger children was −10% (95% posterior credible interval = −19% to −2%). No alternative priors were used but the authors presented Bayesian results alongside results from a standalone frequentist analysis and a pooled frequentist analysis. The estimated difference in the standalone analysis was −18% (95% CI = −36% to 2%). Both Bayesian and standalone analyses concluded non-inferiority for dolutegravir-based therapy, and the Bayesian analysis additionally concluded superiority.

## Conclusion

Our narrative review found that the number of Bayesian methods in confirmatory clinical trials has approximately doubled over the past 6 years, reflecting growing familiarity among investigators. Large confirmatory trials are increasingly viewed as too slow and costly, delaying uptake of effective interventions. This drives interest in flexible adaptive designs that enable smaller, more efficient trials. A Bayesian approach is attractive in this setting, enabling flexible timing of interim analyses and allowing observed data to determine the required sample size, and 47% of trials in our review cited flexibility in adapting the design or use of Bayesian stopping rules as one of their motivations for using a Bayesian approach.

Confirmatory trials are carried out with the aim of informing clinical decisions about which interventions should be used in practice and it is, therefore, important that their results are reliable, accurate and reproducible. For 14% of trials in our review, the priors were not described clearly and it was not possible to determine whether any parameters had been given an informative prior. This is an improvement compared with findings from an earlier review of reporting of Bayesian methods in phase III trials, in which 33% of trials specified no information on the priors used.^26^ However, we repeat the earlier recommendation that priors for all model parameters should be reported, alongside the analysis model and method of implementation, in order that the analysis could be reproduced by others, and that the posterior distribution should be clearly described and interpreted.^27^ As for frequentist analyses, statistical analysis plans (SAPs) should be written in advance, before any data become available.^28^ Results from Bayesian analyses can be sensitive to the priors specified, particularly when the available data are sparse, and it has, therefore, been recommended that sensitivity to chosen priors should be explored.^1^ This was rarely done in trials included in our review; of 15 trials that used informative priors for at least one parameter, only 4 trials also presented a sensitivity analysis using alternative priors.

A limitation of our narrative review is that it included the period following the COVID-19 pandemic, during which many trials evaluating treatments for COVID-19 were carried out, and therefore, the characteristics of the included trials may not be representative of a more typical period of time. Among the COVID-19 trials, 74% mentioned using a flexible adaptive design as a motivation for using Bayesian methods, in comparison with 43% of other infectious disease trials, so this motivating reason is likely to be more prevalent than in other time periods. Our review was narrative rather than systematic: we explored which types of trials have used Bayesian methods and how they were used to identify case studies and issues to consider when deciding whether to use a Bayesian approach. We searched in one database rather than multiple databases and did not include grey literature, so it is likely that some eligible trials were missed. We restricted our review to trials using Bayesian methods for their primary analysis; our findings do not extend to secondary Bayesian analyses.

Among the reasons given for choosing Bayesian methods, we consider the use of informative priors or making probability statements to be the strongest motivations for a Bayesian analysis, because there are no directly equivalent frequentist approaches (Table 3). Making probability statements was one of the most common motivations provided, while informative priors were not often used. Dynamic methods for borrowing information while controlling the type I error rate can increase the acceptability of informative priors in confirmatory trials, and these were used in 4 of the 10 included trials borrowing external information. In settings with recruitment difficulties, such as paediatric trials, rare diseases or subgroups of participants with comorbidities, we recommend considering borrowing relevant information from external sources or within the trial, to gain power and precision. In all confirmatory trial settings, we recommend that Bayesian approaches are used only with careful justification, investigators make clear whether the methods and priors were pre-planned, and alternative frequentist approaches are considered.

## Supplemental Material

sj-docx-1-ctj-10.1177_17407745261437669 – Supplemental material for Bayesian analysis in confirmatory clinical trials: A narrative review and discussion of current practice Supplemental material, sj-docx-1-ctj-10.1177_17407745261437669 for Bayesian analysis in confirmatory clinical trials: A narrative review and discussion of current practice by Rebecca M Turner, Conor D Tweed, Trinh Duong, Deborah Ford, Michelle N Clements, Mahesh KB Parmar, Anna Turkova and Ian R White in Clinical Trials
