# Supplementary material for: Bayesian analysis in confirmatory clinical trials: A narrative review and discussion of current practice
Source: Clin Trials. 2026 Apr 7;23(4):404–12. doi: 10.1177/17407745261437669 (PMC13451585; doi:10.1177/17407745261437669)
Supplement: Supplementary file 1 [file sj-docx-1-ctj-10.1177_17407745261437669.docx]

Bayesian analysis in confirmatory clinical trials: a narrative review and discussion of current practice: supplementary material

## PRISMA flow diagram for narrative review

**Identification of studies**

Records removed before screening:

Duplicate records removed (n = 0)

Records marked as ineligible by automation tools (n = 0)

Records removed for other reasons (n = 0)

Records identified from:

PubMed databases (n = 808)

**Identification**

Records screened

(n = 808)

Records excluded

(n = 616)

Reports sought for retrieval

(n = 192)

Reports not retrieved

(n = 0)

**Screening**

Reports excluded:

Primary analysis was frequentist (n = 34)

Exploratory study (n = 27)

Report of secondary analysis of trial data (n = 11)

No sample size justification (n = 9)

Phase I or II study (n = 7)

Not evaluating a healthcare intervention (n = 3)

Date out of range (n = 3)

Pilot/feasibility study (n = 2)

Design paper (n = 1)

Conference abstract (n = 1)

Reports assessed for eligibility

(n = 192)

Studies included in review

(n = 69)

Reports of included studies

(n = 94)

**Included**

**Reference:**

Page MJ, McKenzie JE, Bossuyt PM, et al. The PRISMA 2020 statement: An updated guideline for reporting systematic reviews. *Int J Surg* 2021; 88. DOI: ARTN 105906 10.1016/j.ijsu.2021.105906

## References for included papers

1.	Adeoye O, Broderick J, Derdeyn CP, et al. Adjunctive Intravenous Argatroban or Eptifibatide for Ischemic Stroke. *New Engl J Med* 2024; 391: 810-820. DOI: 10.1056/NEJMoa2314779.

2.	Alibhai SMH, Papadopoulos E, Mina DS, et al. Home-based versus supervised group exercise in men with prostate cancer on androgen deprivation therapy: A randomized controlled trial and economic analysis. *J Geriatr Oncol* 2024; 15. DOI: ARTN 101646

10.1016/j.jgo.2023.101646.

3.	Amuge P, Lugemwa A, Wynne B, et al. Once-daily dolutegravir-based antiretroviral therapy in infants and children living with HIV from age 4 weeks: results from the below 14 kg cohort in the randomised ODYSSEY trial. *Lancet Hiv* 2022; 9: E638-E648.

4.	Angus DC, Derde L, Al-Beidh F, et al. Effect of Hydrocortisone on Mortality and Organ Support in Patients With Severe COVID-19 The REMAP-CAP COVID-19 Corticosteroid Domain Randomized Clinical Trial. *Jama-J Am Med Assoc* 2020; 324: 1317-1329. DOI: 10.1001/jama.2020.17022.

5.	Arabi YM, Gordon AC, Derde LPG, et al. Lopinavir-ritonavir and hydroxychloroquine for critically ill patients with COVID-19: REMAP-CAP randomized controlled trial. *Intens Care Med* 2021; 47: 867-886. DOI: 10.1007/s00134-021-06448-5.

6.	Barohn RJ, Gajewski B, Pasnoor M, et al. Patient Assisted Intervention for Neuropathy: Comparison of Treatment in Real Life Situations (PAIN-CONTRoLS) Bayesian Adaptive Comparative Effectiveness Randomized Trial. *Jama Neurol* 2021; 78: 68-76. DOI: 10.1001/jamaneurol.2020.2590.

7.	Bendtsen M, Åsberg K and McCambridge J. Effectiveness of a digital intervention versus alcohol information for online help-seekers in Sweden: a randomised controlled trial. *Bmc Med* 2022; 20. DOI: ARTN 176

10.1186/s12916-022-02374-5.

8.	Berger JS, Kornblith LZ, Gong MN, et al. Effect of P2Y12 Inhibitors on Survival Free of Organ Support Among Non-Critically Ill Hospitalized Patients With COVID-19 A Randomized Clinical Trial. *Jama-J Am Med Assoc* 2022; 327: 227-236. DOI: 10.1001/jama.2021.23605.

9.	Berger JS, Neal MD, Kornblith LZ, et al. Effect of P2Y12 Inhibitors on Organ Support-Free Survival in Critically Ill Patients Hospitalized for COVID-19: A Randomized Clinical Trial. *Jama Netw Open* 2023; 6. DOI: ARTN e2314428

10.1001/jamanetworkopen.2023.14428.

10.	Blakely ML, Krzyzaniak A, Dassinger MS, et al. Effect of Early vs Late Inguinal Hernia Repair on Serious Adverse Event Rates in Preterm Infants: A Randomized Clinical Trial. *Jama-J Am Med Assoc* 2024; 331: 1035-1044. DOI: 10.1001/jama.2024.2302.

11.	Böhm M, Kario K, Kandzari DE, et al. Efficacy of catheter-based renal denervation in the absence of antihypertensive medications (SPYRAL HTN-OFF MED Pivotal): a multicentre, randomised, sham-controlled trial. *Lancet* 2020; 395: 1444-1451. DOI: 10.1016/S0140-6736(20)30554-7.

12.	Boulware DR, Lindsell CJ, Stewart TG, et al. Inhaled Fluticasone Furoate for Outpatient Treatment of Covid-19. *N Engl J Med* 2023; 389: 1085-1095. DOI: 10.1056/NEJMoa2209421.

13.	Bradbury CA, Lawler PR, Stanworth SJ, et al. Effect of Antiplatelet Therapy on Survival and Organ Support-Free Days in Critically Ill Patients With COVID-19 A Randomized Clinical Trial. *Jama-J Am Med Assoc* 2022; 327: 1247-1259. DOI: 10.1001/jama.2022.2910.

14.	Brennan B, Kirton L, Marec-Bérard P, et al. Comparison of two chemotherapy regimens in patients with newly diagnosed Ewing sarcoma (EE2012): an open-label, randomised, phase 3 trial. *Lancet* 2022; 400: 1513-1521. DOI: 10.1016/S0140-6736(22)01790-1.

15.	Brückner H, Wallot S, Horvath H, et al. Effectiveness of an online recovery training for employees exposed to blurred boundaries between work and non-work: Bayesian analysis of a randomised controlled trial. *Bmj Mental Health* 2024; 27. DOI: ARTN e301016

10.1136/bmjment-2024-301016.

16.	Butler CC, Dorward J, Yu LM, et al. Azithromycin for community treatment of suspected COVID-19 in people at increased risk of an adverse clinical course in the UK (PRINCIPLE): a randomised, controlled, open-label, adaptive platform trial. *Lancet* 2021; 397: 1063-1074. DOI: 10.1016/S0140-6736(21)00461-X.

17.	Butler CC, Hobbs FR, Gbinigie OA, et al. Molnupiravir plus usual care versus usual care alone as early treatment for adults with COVID-19 at increased risk of adverse outcomes (PANORAMIC): an open-label, platform-adaptive randomised controlled trial. *Lancet* 2023; 401: 281-293. DOI: 10.1016/S0140-6736(22)02597-1.

18.	Butler CC, van der Velden AW, Bongard E, et al. Oseltamivir plus usual care versus usual care for influenza-like illness in primary care: an open-label, pragmatic, randomised controlled trial. *Lancet* 2020; 395: 42-52. DOI: 10.1016/S0140-6736(19)32982-4.

19.	Butler CC, Yu LM, Dorward J, et al. Doxycycline for community treatment of suspected COVID-19 in people at high risk of adverse outcomes in the UK (PRINCIPLE): a randomised, controlled, open-label, adaptive platform trial. *Lancet Resp Med* 2021; 9: 1010-1020. DOI: 10.1016/S2213-2600(21)00310-6.

20.	Cameron MH, Hildebrand A, Hugos CL, et al. Free From Falls education and exercise program for reducing falls in people with multiple sclerosis: A randomized controlled trial. *Mult Scler J* 2022; 28: 980-988. DOI: Artn 13524585211046898

10.1177/13524585211046898.

21.	Chamberlain JM, Kapur J, Shinnar S, et al. Efficacy of levetiracetam, fosphenytoin, and valproate for established status epilepticus by age group (ESETT): a double-blind, responsive-adaptive, randomised controlled trial. *Lancet* 2020; 395: 1217-1224. DOI: 10.1016/S0140-6736(20)30611-5.

22.	Chaumeau V, Kajeechiwa L, Kulabkeeree T, et al. Outdoor residual spraying for malaria vector-control in Kayin (Karen) state, Myanmar: A cluster randomized controlled trial. *Plos One* 2022; 17. DOI: ARTN e0274320

10.1371/journal.pone.0274320.

23.	Clairman H, Dover S, Tomlinson G, et al. Lengthening sleep reduces pain in childhood arthritis: a crossover randomised controlled trial. *Rmd Open* 2023; 9. DOI: ARTN e003352

10.1136/rmdopen-2023-003352.

24.	Collins SP, Shotwell MS, Strich JR, et al. Fostamatinib for Hospitalized Adults With COVID-19 and Hypoxemia A Randomized Clinical Trial. *Jama Netw Open* 2024; 7. DOI: ARTN e2448215

10.1001/jamanetworkopen.2024.48215.

25.	Criner GJ, Delage A, Voelker K, et al. Improving Lung Function in Severe Heterogenous Emphysema with the Spiration Valve System (EMPROVE) A Multicenter, Open-Label Randomized Controlled Clinical Trial. *Am J Resp Crit Care* 2019; 200: 1354-1362. DOI: 10.1164/rccm.201902-0383OC.

26.	De Laere B, Crippa A, Discacciati A, et al. Androgen receptor pathway inhibitors and taxanes in metastatic prostate cancer: an outcome-adaptive randomized platform trial. *Nat Med* 2024; 30: 3291-3302. DOI: 10.1038/s41591-024-03204-2.

27.	Dieussaert I, Kim JH, Luik S, et al. RSV Prefusion F Protein-Based Maternal Vaccine - Preterm Birth and Other Outcomes. *New Engl J Med* 2024; 390: 1009-1021. DOI: 10.1056/NEJMoa2305478.

28.	Dorward J, Yu LM, Hayward G, et al. Colchicine for COVID-19 in the community (PRINCIPLE): a randomised, controlled, adaptive platform trial. *Brit J Gen Pract* 2022. DOI: 10.3399/Bjgp.2022.0083.

29.	Edwards DJ, Liu CY, Dunning K, et al. Electric Field Navigated 1-Hz rTMS for Poststroke Motor Recovery: The E-FIT Randomized Controlled Trial. *Stroke* 2023; 54: 2254-2264. DOI: 10.1161/Strokeaha.123.043164.

30.	Estcourt LJ, Turgeon AF, McQuilten ZK, et al. Effect of Convalescent Plasma on Organ Support-Free Days in Critically Ill Patients With COVID-19 A Randomized Clinical Trial. *Jama-J Am Med Assoc* 2021; 326: 1690-1702. DOI: 10.1001/jama.2021.18178.

31.	Fakes K, Waller A, Carey M, et al. Discharge intervention to improve outcomes and web-based portal engagement after stroke and transient ischaemic attack: A randomised controlled trial. *J Stroke Cerebrovasc* 2024; 33. DOI: ARTN 107771

10.1016/j.jstrokecerebrovasdis.2024.107771.

32.	Felten R, Devauchelle-Pensec V, Seror R, et al. Interleukin 6 receptor inhibition in primary Sjogren syndrome: a multicentre double-blind randomised placebo-controlled trial. *Ann Rheum Dis* 2021; 80: 329-338. DOI: 10.1136/annrheumdis-2020-218467.

33.	Ferreira D, Hardy J, Meere W, et al. Fasting vs. no fasting prior to catheterization laboratory procedures: the SCOFF trial. *Eur Heart J* 2024. DOI: 10.1093/eurheartj/ehae573.

34.	Foley MJ, Rajkumar CA, Ahmed-Jushuf F, et al. Coronary sinus reducer for the treatment of refractory angina (ORBITA-COSMIC): a randomised, placebo-controlled trial. *Lancet* 2024; 403: 1543-1553. DOI: 10.1016/S0140-6736(24)00256-3.

35.	Gharbharan A, Jordans C, Zwaginga L, et al. Original Outpatient convalescent plasma therapy for high-risk patients with early COVID-19: a randomized placebo-controlled trial. *Clin Microbiol Infec* 2023; 29: 208-214. DOI: 10.1016/j.cmi.2022.08.005.

36.	Gibson CM, Holmes D, Mikdadi G, et al. Implantable Cardiac Alert System for Early Recognition of ST-Segment Elevation Myocardial Infarction. *J Am Coll Cardiol* 2019; 73: 1919-1927. DOI: 10.1016/j.jacc.2019.01.014.

37.	Goligher EC, Bradbury CA, McVerry BJ, et al. Therapeutic Anticoagulation with Heparin in Critically Ill Patients with Covid-19. *New Engl J Med* 2021; 385: 777-789. DOI: 10.1056/NEJMoa2103417.

38.	Gordon AC, Al-Beidh F, Rowan KM, et al. Interleukin-6 Receptor Antagonists in Critically Ill Patients with Covid-19. *New Engl J Med* 2021; 384: 1491-1502. DOI: 10.1056/NEJMoa2100433.

39.	Group RC. Immunomodulatory therapy in children with paediatric inflammatory multisystem syndrome temporally associated with SARS-CoV-2 (PIMS-TS, MIS-C; RECOVERY): a randomised, controlled, open-label, platform trial. *Lancet Child Adolesc Health* 2024; 8: 190-200. 20240122. DOI: 10.1016/S2352-4642(23)00316-4.

40.	Hayward G, Yu LM, Little P, et al. Ivermectin for COVID-19 in adults in the community (PRINCIPLE): An open, randomised, controlled, adaptive platform trial of short- and longer-term outcomes. *J Infection* 2024; 88. DOI: ARTN 106130

10.1016/j.jinf.2024.106130.

41.	Hills TE, Lorenzi E, Berry LR, et al. Simvastatin in Critically Ill Patients with Covid-19. *New Engl J Med* 2023; 389: 2341-2354. DOI: 10.1056/NEJMoa2309995.

42.	Hobbs FR, Gbinigie-Thompson OA, Shanyinde M, et al. Favipiravir for COVID-19 in adults in the community in PRINCIPLE, an open-label, randomised, controlled, adaptive platform trial of short- and longer-term outcomes. *J Infection* 2024; 89. DOI: ARTN 106248

10.1016/j.jinf.2024.106248.

43.	Huang DT, McCreary EK, Bariola JR, et al. Effectiveness of Casirivimab-Imdevimab and Sotrovimab During a SARS-CoV-2 Delta Variant Surge: A Cohort Study and Randomized Comparative Effectiveness Trial. *Jama Netw Open* 2022; 5: e2220957. 20220701. DOI: 10.1001/jamanetworkopen.2022.20957.

44.	Iglesias JF, Muller O, Heg D, et al. Biodegradable polymer sirolimus-eluting stents versus durable polymer everolimus-eluting stents in patients with ST-segment elevation myocardial infarction (BIOSTEMI): a single-blind, prospective, randomised superiority trial. *Lancet* 2019; 394: 1243-1253. DOI: 10.1016/S0140-6736(19)31877-X.

45.	Iglesias JF, Roffi M, Losdat S, et al. Long-term outcomes with biodegradable polymer sirolimus- eluting stents versus durable polymer everolimus-eluting stents in ST-segment elevation myocardial infarction: 5-year follow-up of the BIOSTEMI randomised superiority trial. *Lancet* 2023; 402: 1979-1990. DOI: 10.1016/S0140-6736(23)02197-9.

46.	Jardine MJ, Kotwal SS, Bassi A, et al. Angiotensin receptor blockers for the treatment of covid-19: pragmatic, adaptive, multicentre, phase 3, randomised controlled trial. *Bmj-Brit Med J* 2022; 379. DOI: ARTN e072175

10.1136/bmj-2022-072175.

47.	Kandzari DE, Townsend RR, Kario K, et al. Safety and Efficacy of Renal Denervation in Patients Taking Antihypertensive Medications. *Jacc-Journal of the American College of Cardiology* 2023; 82: 1809-1823. DOI: 10.1016/j.jacc.2023.08.045.

48.	Kapur J, Elm J, Chamberlain JM, et al. Randomized Trial of Three Anticonvulsant Medications for Status Epilepticus. *New Engl J Med* 2019; 381: 2103-2113. DOI: 10.1056/NEJMoa1905795.

49.	Karno MP, Rawson R, Rogers B, et al. Effect of screening, brief intervention and referral to treatment for unhealthy alcohol and other drug use in mental health treatment settings: a randomized controlled trial. *Addiction* 2021; 116: 159-169. DOI: 10.1111/add.15114.

50.	Kerem E, Cohen-Cymberknoh M, Tsabari R, et al. Ivacaftor in People with Cystic Fibrosis and a 3849+10kb C→T or D1152H Residual Function Mutation. *Ann Am Thorac Soc* 2021; 18: 433-441. DOI: 10.1513/AnnalsATS.202006-659OC.

51.	Khanna S, Assi M, Lee C, et al. Efficacy and Safety of RBX2660 in PUNCH CD3, a Phase III, Randomized, Double-Blind, Placebo-Controlled Trial with a Bayesian Primary Analysis for the Prevention of Recurrent

Infection. *Drugs* 2022; 82: 1527-1538. DOI: 10.1007/s40265-022-01797-x.

52.	Kinoshita T, Shinoda M, Nishizaki Y, et al. A multicenter, double-blind, randomized, parallel-group, placebo-controlled study to evaluate the efficacy and safety of camostat mesilate in patients with COVID-19 (CANDLE study). *Bmc Med* 2022; 20. DOI: ARTN 342

10.1186/s12916-022-02518-7.

53.	Korley FK, Durkalski-Mauldin V, Yeatts SD, et al. Early Convalescent Plasma for High-Risk Outpatients with Covid-19. *New Engl J Med* 2021; 385: 1951-1960. DOI: 10.1056/NEJMoa2103784.

54.	Kosiborod MN, Windsor SL, Vardeny O, et al. Effect of sodium-glucose co-transporter-2 inhibitors on survival free of organ support in patients hospitalised for COVID-19 (ACTIV-4a): a pragmatic, multicentre, open-label, randomised, controlled, platform trial. *Lancet Diabetes Endo* 2024; 12: 725-734. DOI: 10.1016/S2213-8587(24)00218-3.

55.	Lakkireddy DR, Wilber DJ, Mittal S, et al. Pulmonary Vein Isolation With or Without Left Atrial Appendage Ligation in Atrial Fibrillation: The aMAZE Randomized Clinical Trial. *Jama-J Am Med Assoc* 2024; 331: 1099-1108. DOI: 10.1001/jama.2024.3026.

56.	Lane C, Wolfenden L, Hall A, et al. Optimising a multi-strategy implementation intervention to improve the delivery of a school physical activity policy at scale: findings from a randomised noninferiority trial. *Int J Behav Nutr Phy* 2022; 19. DOI: ARTN 106

10.1186/s12966-022-01345-6.

57.	Lawler PR, Derde LPG, van de Veerdonk FL, et al. Effect of Angiotensin-Converting Enzyme Inhibitor and Angiotensin Receptor Blocker Initiation on Organ Support-Free Days in Patients Hospitalized With COVID-19 A Randomized Clinical Trial. *Jama-J Am Med Assoc* 2023; 329: 1183-1196. DOI: 10.1001/jama.2023.4480.

58.	Lawler PR, Golighe EC, Berge JS, et al. Therapeutic Anticoagulation with Heparin in Noncritically Ill Patients with Covid-19. *New Engl J Med* 2021; 385: 790-802. DOI: 10.1056/NEJMoa2105911.

59.	Mariette X, Hermine O, Tharaux PL, et al. Effect of anakinra versus usual care in adults in hospital with COVID-19 and mild-to-moderate pneumonia (CORIMUNO-ANA-1): a randomised controlled trial. *Lancet Resp Med* 2021; 9: 295-304. DOI: 10.1016/S2213-2600(20)30556-7.

60.	McCreary EK, Bariola JR, Minnier TE, et al. The comparative effectiveness of COVID-19 monoclonal antibodies: A learning health system randomized clinical trial. *Contemp Clin Trials* 2022; 119. DOI: ARTN 106822

10.1016/j.cct.2022.106822.

61.	McNeely J, Wang SS, Abadi YR, et al. Addiction Consultation Services for Opioid Use Disorder Treatment Initiation and Engagement. *Jama Intern Med* 2024; 184: 1106-1115. DOI: 10.1001/jamainternmed.2024.3422.

62.	Michielsens CAJ, den Broeder N, van den Hoogen FHJ, et al. Treat-to-target dose reduction and withdrawal strategy of TNF inhibitors in psoriatic arthritis and axial spondyloarthritis: a randomised controlled non-inferiority trial. *Ann Rheum Dis* 2022; 81: 1392-1399. DOI: 10.1136/annrheumdis-2022-222260.

63.	Morpeth SC, Venkatesh B, Totterdell JA, et al. A Randomized Trial of Nafamostat for Covid-19. *Nejm Evid* 2023; 2. DOI: ARTN EVIDoa2300132

10.1056/EVIDoa2300132.

64.	Mosnaim GS, Hoyte FCL, Safioti G, et al. Effectiveness of a Maintenance and Reliever Digihaler System in Asthma: 24-Week Randomized Study (CONNECT2). *J Aller Cl Imm-Pract* 2024; 12. DOI: 10.1016/j.jaip.2023.11.037.

65.	Naggie S, Boulware DR, Lindsell CJ, et al. Effect of Ivermectin vs Placebo on Time to Sustained Recovery in Outpatients With Mild to Moderate COVID-19 A Randomized Clinical Trial. *Jama-J Am Med Assoc* 2022; 328: 1595-1603. DOI: 10.1001/jama.2022.18590.

66.	Nooijen CFJ, Blom V, Ekblom Ö, et al. The effectiveness of multi-component interventions targeting physical activity or sedentary behaviour amongst office workers: a three-arm cluster randomised controlled trial. *Bmc Public Health* 2020; 20. DOI: ARTN 1329

10.1186/s12889-020-09433-7.

67.	Oreskovic T, Percac-Lima S, Ashburner JM, et al. Cytisine Versus Varenicline for Smoking Cessation in a Primary Care Setting: A Randomized Non-inferiority Trial. *Nicotine Tob Res* 2023; 25: 1547-1555. DOI: 10.1093/ntr/ntad065.

68.	Ortigoza MB, Yoon H, Goldfeld KS, et al. Efficacy and Safety of COVID-19 Convalescent Plasma in Hospitalized Patients A Randomized Clinical Trial. *Jama Intern Med* 2022; 182: 115-126. DOI: 10.1001/jamainternmed.2021.6850.

69.	Perri MG, Shankar MN, Daniels MJ, et al. Effect of Telehealth Extended Care for Maintenance of Weight Loss in Rural US Communities A Randomized Clinical Trial. *Jama Netw Open* 2020; 3. DOI: ARTN e206764

10.1001/jamanetworkopen.2020.6764.

70.	Pilgrim T, Muller O, Heg D, et al. Biodegradable- Versus Durable-Polymer Drug-Eluting Stents for STEMI: Final 2-Year Outcomes of the BIOSTEMI Trial. *JACC Cardiovasc Interv* 2021; 14: 639-648. DOI: 10.1016/j.jcin.2020.12.011.

71.	Pineles BL, Vial M, Castro T, et al. Ambulation for latency during expectant management of preterm prelabor rupture of membranes: a randomized controlled trial (AMBLE). *Am J Obst Gynec Mfm* 2024; 6. DOI: ARTN 101218

10.1016/j.ajogmf.2023.101218.

72.	Polack FP, Thomas SJ, Kitchin N, et al. Safety and Efficacy of the BNT162b2 mRNA Covid-19 Vaccine. *New Engl J Med* 2020; 383: 2603-2615. DOI: 10.1056/NEJMoa2034577.

73.	Popma JJ, Deeb GM, Yakubov SJ, et al. Transcatheter Aortic-Valve Replacement with a Self-Expanding Valve in Low-Risk Patients. *New Engl J Med* 2019; 380: 1706-1715. DOI: 10.1056/NEJMoa1816885.

74.	Pradilla G, Ratcliff JJ, Hall AJ, et al. Trial of Early Minimally Invasive Removal of Intracerebral Hemorrhage. *New Engl J Med* 2024; 390: 1277-1289. DOI: 10.1056/NEJMoa2308440.

75.	Pua YH, Yeo SJ, Clark RA, et al. Cost and outcomes of Hospital-based Usual cAre versus Tele-monitor self-directed Rehabilitation (HUATR) in patients with total knee arthroplasty: A randomized, controlled, non-inferiority trial. *Osteoarthr Cartilage* 2024; 32: 601-611. DOI: 10.1016/j.joca.2023.11.017.

76.	Rampon G, Jia SJ, Agrawal R, et al. Smartphone-Guided Self-prone Positioning vs Usual Care in Nonintubated Hospital Ward Patients With COVID-19 A Pragmatic Randomized Clinical Trial. *Chest* 2022; 162: 782-791. DOI: 10.1016/j.chest.2022.05.009.

77.	Reddy VY, Gerstenfeld EP, Natale A, et al. Pulsed Field or Conventional Thermal Ablation for Paroxysmal Atrial Fibrillation. *New Engl J Med* 2023; 389: 1660-1671. DOI: 10.1056/NEJMoa2307291.

78.	Reis G, Moreira-Silva EAD and Silva DCM. Effect of early treatment with fluvoxamine on risk of emergency care and hospitalisation among patients with COVID-19: the TOGETHER randomised, platform clinical trial (vol 10, pg e42, 2021). *Lancet Glob Health* 2022; 10: E481-E481. DOI: 10.1016/S2214-109x(22)00081-X.

79.	Reis G, Silva EADM, Silva DCM, et al. Oral Fluvoxamine With Inhaled Budesonide for Treatment of Early-Onset COVID-19 A Randomized Platform Trial. *Ann Intern Med* 2023; 176: 667-+. DOI: 10.7326/M22-3305.

80.	Reis G, Silva EADM, Silva DCM, et al. Effect of spirulina on risk of hospitalization among patients with COVID-19: the TOGETHER randomized trial. *Am J Clin Nutr* 2024; 120: 602-609. DOI: 10.1016/j.ajcnut.2024.06.016.

81.	Reis G, Silva EAM, Silva DCM, et al. Early Treatment with Pegylated Interferon Lambda for Covid-19. *New Engl J Med* 2023; 388: 518-528. DOI: 10.1056/NEJMoa2209760.

82.	Reis G, Silva EASM, Silva DCM, et al. Effect of Early Treatment with Ivermectin among Patients with Covid-19. *New Engl J Med* 2022; 386: 1721-1731. DOI: 10.1056/NEJMoa2115869.

83.	Richter KP, Catley D, Gajewski BJ, et al. The Effects of Opt-out vs Opt-in Tobacco Treatment on Engagement, Cessation, and Costs A Randomized Clinical Trial. *Jama Intern Med* 2023; 183: 331-339. DOI: 10.1001/jamainternmed.2022.7170.

84.	Rotheram-Borus MJ, Tomlinson M, Worthman CM, et al. Maternal depression, alcohol use, and transient effects of perinatal paraprofessional home visiting in South Africa: Eight-year follow-up of a cluster randomized controlled trial. *Soc Sci Med* 2023; 324. DOI: ARTN 115853

10.1016/j.socscimed.2023.115853.

85.	Rothman RL, Stewart TG, Mourad A, et al. Time to Sustained Recovery Among Outpatients With COVID-19 Receiving Montelukast vs Placebo. *Jama Netw Open* 2024; 7. DOI: ARTN e2439332

10.1001/jamanetworkopen.2024.39332.

86.	Solomon SD, Lowenstein CJ, Bhatt AS, et al. Effect of the P-Selectin Inhibitor Crizanlizumab on Survival Free of Organ Support in Patients Hospitalized for COVID-19: A Randomized Controlled Trial. *Circulation* 2023; 148: 381-390. DOI: 10.1161/Circulationaha.123.065190.

87.	Stahl A, Sukgen EA, Wu WC, et al. Effect of Intravitreal Aflibercept vs Laser Photocoagulation on Treatment Success of Retinopathy of Prematurity The FIREFLEYE Randomized Clinical Trial. *Jama-J Am Med Assoc* 2022; 328: 348-359. DOI: 10.1001/jama.2022.10564.

88.	ten Doesschate T, van der Vaart TW, Debisarun PA, et al. Bacillus Calmette-Guerin vaccine to reduce healthcare worker absenteeism in COVID-19 pandemic, a randomized controlled trial. *Clin Microbiol Infec* 2022; 28: 1278-1285. DOI: 10.1016/j.cmi.2022.04.009.

89.	Thomas HMM, Enkel SL, Mullane M, et al. Trimodal skin health programme for childhood impetigo control in remote Western Australia (SToP): a cluster randomised, stepped-wedge trial. *Lancet Child Adolesc* 2024; 8: 809-820. DOI: 10.1016/S2352-4642(24)00229-3.

90.	Wegener ST, Vallier HA, Huang YJ, et al. Early Effects of the Trauma Collaborative Care Intervention: Results From a Prospective Multicenter Cluster Clinical Trial. *J Orthop Trauma* 2019; 33: 538-546. DOI: 10.1097/Bot.0000000000001581.

91.	Witte SS, Pala AN, Mukherjee TI, et al. Reducing Partner Violence Against Women who Exchange Sex and use Drugs through a Combination Microfinance and HIV Risk Reduction Intervention: A Cluster Randomized Trial. *Aids Behav* 2023; 27: 4084-4093. DOI: 10.1007/s10461-023-04122-z.

92.	Wong JYH, Zhang W, Wu YD, et al. An Interactive Web-Based Sexual Health Literacy Program for Safe Sex Practice for Female Chinese University Students: Multicenter Randomized Controlled Trial. *J Med Internet Res* 2021; 23. DOI: ARTN e22564

10.2196/22564.

93.	Yorozuya K, Kubo Y, Asaoka Y, et al. A multimodal non-pharmacological intervention for persons with dementia in nursing homes: a multicentre randomised controlled trial. *Psychogeriatrics* 2024; 24: 1255-1266. DOI: 10.1111/psyg.13186.

94.	Yu LM, Bafadhel M, Dorward J, et al. Inhaled budesonide for COVID-19 in people at high risk of complications in the community in the UK (PRINCIPLE): a randomised, controlled, open-label, adaptive platform trial. *Lancet* 2021; 398: 843-855. DOI: 10.1016/S0140-6736(21)01744-X.
